# Polarity-tunable dye-sensitized optoelectronic artificial synapses for physical reservoir computing-based machine vision

**DOI:** 10.1038/s41598-025-00693-0

**Published:** 2025-05-12

**Authors:** Hiroaki Komatsu, Norika Hosoda, Takashi Ikuno

**Affiliations:** Department of Applied Electronics, Graduate School of Advanced Engineering, Tokyo University of Science, Katsushika, Tokyo, 125-8585 Japan

**Keywords:** Optoelectronic artificial synapse, self-powered, dye-sensitized solar cell, physical reservoir computing, Electronic devices, Sensors and biosensors, Electronic properties and materials

## Abstract

Conventional machine vision systems process huge time-series data per second, presenting significant challenges for edge-device applications due to limitations in data transfer and storage. Inspired by the human visual system, artificial optoelectronic synapses replicating synaptic responses have emerged as promising solutions. However, achieving color recognition comparable to human vision remains challenging. Moreover, most optoelectronic artificial synapses rely on photocurrent-based operation, producing low current values and necessitating external circuits. This study reports a self-powered optoelectronic artificial synapse capable of distinguishing wavelengths with a resolution of 10 nm by integrating dye-sensitized solar cells. The device exhibits synaptic responses to light pulses and bipolar responses when exposed to different wavelengths. The wavelength-dependent bipolar behavior enables exceptional separation capabilities, achieving six-bit resolution with 64 distinct states and supporting multiple logic operations, including AND, OR, and XOR, within a single device. Additionally, the device leverages distinct responses to red, green, and blue light irradiation for physical reservoir computing, facilitating the classification of color-coded human motion with an accuracy of 82%. These findings advance the development of optoelectronic artificial synapses for precise, human-eye-like color discrimination.

**Supplementary Information:**

The online version contains supplementary material available at 10.1038/s41598-025-00693-0.

## Introduction

Conventional silicon-based machine vision systems convert time-series light data collected through a lens into electrical signals via photosensors, which are subsequently stored in memory for computational processing^1^ These systems typically operate at 10–60 frames per second (FPS), requiring frequent sampling and conversion of light data into electrical signals^2^ Although this enables high-resolution data acquisition, it demands substantial storage and computational resources, limiting deployment in edge-computing environments with restricted power availability. Therefore, developing a power-efficient machine vision system optimized for edge applications is essential.

The human vision system represents an efficient, low-power architecture capable of operating in edge environments^3^ While it cannot record all visual information as accurately as conventional machine vision systems, it excels in cognitive tasks such as object recognition and spatial perception, which are either computationally intensive or challenging for conventional approaches^4^ Mimicking the human visual system offers the potential to execute cognitive tasks with significantly lower power consumption. In biological vision, time-series light data received by the eye are converted into electrical signals in the receptors and transmitted to the brain through synapses in the visual cortex^5^ Studies indicate that not all electrical signals are relayed to the brain; the synapses selectively filter and discard certain signals^6,7^ This synaptic compression of information enhances efficiency and reduces power consumption in the human visual system. Inspired by this mechanism, optoelectronic artificial synapses replicating synaptic behavior have gained attention as foundational components for next-generation power-efficient machine vision systems^8^.

Artificial optoelectronic synapses have been developed based on semiconductor materials such as ZnO, Ga₂O₃, and MoS₂^9-11^ However, achieving color recognition remains a major challenge in artificial optoelectronic synapses. The ability to distinguish light wavelengths, akin to human vision, enhances the extraction of detailed information from visual systems. For instance, color vision offers significant advantages over monochromatic vision in object recognition. Additionally, optical sensors utilizing multiple wavelengths enable high-precision measurements of biological signals, including SpO₂ and heart rate^12,13^ Consequently, developing wavelength-sensitive artificial optoelectronic synapses is highly desirable.

One approach to achieving wavelength sensitivity involves designing artificial synapses with selective responses to specific wavelengths. Hao et al. developed artificial optoelectronic synapses exhibiting wavelength selectivity through organic semiconductors^14^ Their device successfully discriminated input light wavelengths of 365, 450, 520, 620, and 850 nm. Similarly, Islam et al. demonstrated that MoS₂ transistors with PtTe₂/Si gate electrodes could distinguish between wavelengths of 300, 450, 1000, and 2000 nm^15^ However, a key limitation of this approach is the reliance on constant light intensity. Variations in intensity compromise the ability of the device to accurately reflect wavelength-dependent sensitivity, impairing wavelength discrimination.

An alternative strategy involves designing devices that generate outputs with opposite polarities depending on the incident light wavelength. Wavelength-dependent polarity switching enables straightforward wavelength identification, even under fluctuating light intensities. Several studies have reported devices capable of modulating photoconductivity based on the incident wavelength^16-20^ For instance, Ahmed et al. demonstrated wavelength-dependent bipolar responses in black phosphorus, attributed to trap sites induced by oxygen absorption, effectively distinguishing between ultraviolet (UV)-A and UV-B light^21^ Ge et al. observed similar bipolar responses in a perovskite-ZnO heterostructure, where changes in depletion layer width under 365 nm and 520 nm illumination enabled wavelength discrimination^22^ Additionally, Zhang et al. reported that MoS₂-based field-effect transistors (FETs) exhibit bipolar responses to visible and infrared (IR) light due to the photogating effect^23^ These devices primarily exhibit wavelength-dependent bipolar behavior when the wavelength regions differ significantly, such as between UV and visible light or between visible and IR light^23-26^ No studies have demonstrated an optoelectronic artificial synapse exhibiting bipolar behavior within the visible light spectrum.

Most reported optoelectronic artificial synapses operate based on the photocurrent mechanism^15,23,25,27^ Photocurrent-based devices require an applied voltage, resulting in high energy consumption while generating relatively small currents in the nanoampere to microampere range. Although photovoltaic-based optoelectronic artificial synapses have been explored, these devices predominantly rely on PN junctions, which are constrained by low photovoltages of approximately 100 mV^14^.

To address these challenges, dye-sensitized solar cells (DSCs) have been proposed as promising candidates for self-powered optoelectronic artificial synapses^28-31^ This is because DSCs can exhibit synaptic responses to temporal variations in light intensity, owing to their ability to significantly alter the time constant of photovoltage while maintaining a nearly constant open-circuit voltage under varying light intensities. Furthermore, their high wavelength sensitivity, attributed to the discrete energy levels of dyes, provides a distinct advantage over conventional semiconductors that rely on band-edge absorption for wavelength discrimination. Therefore, we believe DSCs represent a suitable platform for wavelength-sensitive optoelectronic artificial synapses^28^.

This study presents a self-powered, polarity-tunable dye-sensitized optoelectronic artificial synapse integrating DSCs sensitized with two dyes. The device exhibited bipolar photovoltage responses across the 450–750 nm wavelength range, enabling wavelength discrimination with a 10 nm precision. The device was employed within a physical reservoir computing (PRC) framework to evaluate its capability for processing time-series data, leveraging its intrinsic material dynamics for machine learning tasks^32,33^ The concept of PRC is to reduce training costs by transforming time-series data through a material and training only a lightweight output layer, such as a linear regression model. In our PRC systems, we used a single-layer neural network (NN) with an activation function as the output layer to enable multi-class classification. While this design introduces a nonlinearity, the trainable part remains compact and limited to the output stage, preserving the fundamental principle of PRC while enhancing classification performance. The system successfully classified input pulses of up to six bits. The device achieved 82% accuracy in a classification task involving 18 input combinations comprising three colors and six types of motion.

## Results and discussion

**Fig. 1:**
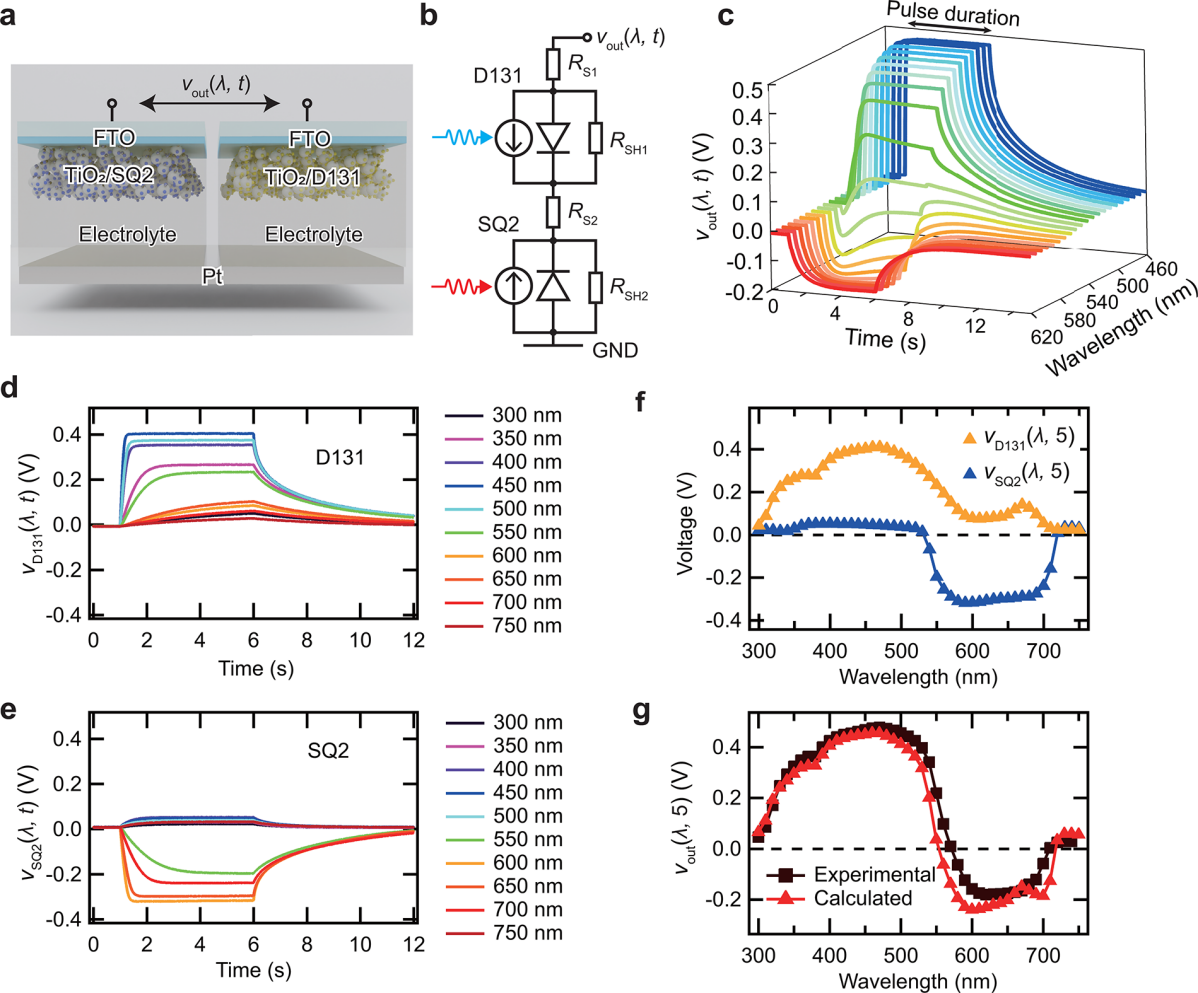
(a) Schematic representation of a self-powered polarity-tunable dye-sensitized optoelectronic artificial synapse. The 3D illustration was created using Blender 4.2.1 LTS (https://www.blender.org/ ). (b) Equivalent circuit of the device. (c) Typical *v*_out_ response induced by a monochromatic light pulse (λ = 460–620 nm, P = 2–2.5 mW). (d) Typical *v*_D131_ response and (e) *v*_SQ2_ response induced by a monochromatic light pulse (λ = 300–750 nm, P = 2–2.5 mW). (f) Steady-state values of *v*_D131_ and *v*_SQ2_ under light illumination as a function of wavelength (λ = 300–750 nm, P = 2–2.5 mW). (g) Comparison of steady-state *v*_out_ values obtained from Eq. (1) and experimental results as a function of wavelength (λ = 300–750 nm, P = 2–2.5 mW).﻿﻿

Figure 1a presents a schematic of the device structure. The device comprised two DSCs sensitized with SQ2 and D131, incorporating common Pt electrodes. The photovoltage (*v*_out_) was measured between the fluorine-doped tin oxide (FTO) electrode of the SQ2-sensitized DSC and the FTO electrode of the D131-sensitized DSC. Figure 1b illustrates the equivalent circuit of the device, where *R*_S1_ and *R*_S2_ denote the series resistances, and *R*_SH1_ and *R*_SH2_ represent the shunt resistances of the DSCs sensitized with D131 and SQ2, respectively. These equivalent circuits indicate that the device connects two DSCs in a back-to-back configuration.

We measured the *v*_out_ responses induced by monochromatic light pulses in the range of 460–620 nm to investigate the photoresponse characteristics as a function of wavelength, as illustrated in Fig. 1c. The pulse duration was set to 5 s. In the 460–550 nm range, *v*_out_ monotonically increased, saturating at a maximum value of 0.48 V under the light pulse. Conversely, *v*_out_monotonically decreased and saturated at a minimum value of -0.18 V under the light pulse for wavelengths above 600 nm. Notably, the response did not exhibit monotonic behavior in the intermediate-wavelength region, where *v*_out_ transitions between positive and negative voltage responses. These results confirm that the device exhibits wavelength-dependent bipolar responses, generating both positive and negative *v*_out_ values depending on the light wavelength, with a resolution of approximately 10 nm.

The individual photoresponses of D131 (*v*_D131_) and SQ2 (*v*_SQ2_) were investigated under monochromatic light pulses spanning from 300 to 750 nm to further understand the origin of the wavelength-dependent bipolar responses of *v*_out_ (see Fig. 1d, e). *v*_D131_ and *v*_SQ2_ were measured by selectively irradiating the respective DSCs with a mask. *v*_SQ2_ and *v*_D131_ represent the photovoltage responses during selective irradiation of the DSCs with SQ2 and D131, respectively. *v*_D131_ exhibited a positive voltage, reaching up to 0.40 V at 450 nm. However, *v*_SQ2_ demonstrated a negative voltage, reaching up to -0.32 V at 600 nm. Figure 1f displays the steady-state voltage values under illumination as a function of wavelength. The steady-state voltage was defined as the voltage measured 5 s after initiating light irradiation. The wavelength regions that exhibit positive and negative *v*_out_ values for D131 and SQ2 correspond with their respective absorption wavelengths, as observed in the incident photon-to-current conversion efficiency (IPCE) spectra (see **Figure ****S1**, Supplementary Information).

*v*_out_ is expected to be the sum of *v*_D131_ and *v*_SQ2_ when both DSCs are illuminated, as described by the following equation:

$$\:\begin{array}{c}{v}_{\text{o}\text{u}\text{t}}\left(\lambda\:,t\right)={v}_{\text{D}131}\left(\lambda\:,t\right)+{v}_{\text{S}\text{Q}2}\left(\lambda\:,t\right).\:\end{array}$$

Figure 1g presents the steady-state value of *v*_out_, calculated from Eq. (1), alongside the experimental results as a function of wavelength, ranging from 300 to 750 nm. The measured *v*_out_ increases with wavelength, reaching a maximum at 470 nm, then decreases rapidly, crossing zero at 570 nm and reaching a minimum at 620 nm. Similarly, the calculated *v*_out_ peaked at 470 nm, then decreased sharply, crossing zero at 550 nm and reaching a minimum at 600 nm. The results obtained using Eq. (1) are consistent with the experimental findings, indicating that the unique properties of the device arise from the competition between *v*_D131_ and *v*_SQ2_.  

Equation (1) also considers the nonmonotonic behavior of *v*_out_ observed during light pulse irradiation at the crossover wavelength, where the absorption spectra of D131 and SQ2 overlap. *v*_out_ under 570 nm light pulse, where the absorption spectra of SQ2 and D131 overlapped, initially shifted in the negative direction but eventually shifted to the positive direction (see **Figure S2a**, Supplementary Information). **Figure S2b** illustrates the transient responses of *v*_D131_ and *v*_SQ2_ to a 570 nm light pulse. *v*_SQ2_ was saturated earlier due to its higher spectral sensitivity at 570 nm compared to D131 (see **Figure ****S1**, Supplementary Information). The sum of *v*_D131_ and *v*_SQ2_ aligned with the *v*_out_ response to the 570 nm light pulse (see **Figure S2c**, Supplementary Information). This behavior suggests that the combined responses of D131 and SQ2 are responsible for the observed nonmonotonicity. Initially, *v*_SQ2_ dominated, leading to a negative shift due to its spectral properties. As *v*_SQ2_ saturated, *v*_D131_ predominated, resulting in a positive shift and accounting for the nonmonotonic behavior of *v*_out_.

This bipolar response induced by light wavelength suggests that the steady-state *v*_out_ can be used for wavelength discrimination. This feature is particularly effective between 450 and 610 nm. Outside this range, different wavelengths may yield the same voltage, making color discrimination difficult. However, since the wavelengths corresponding to colors perceived by the human eye—such as red, blue, and green—fall within this range, the feature is useful for vision systems.

**Fig. 2:**
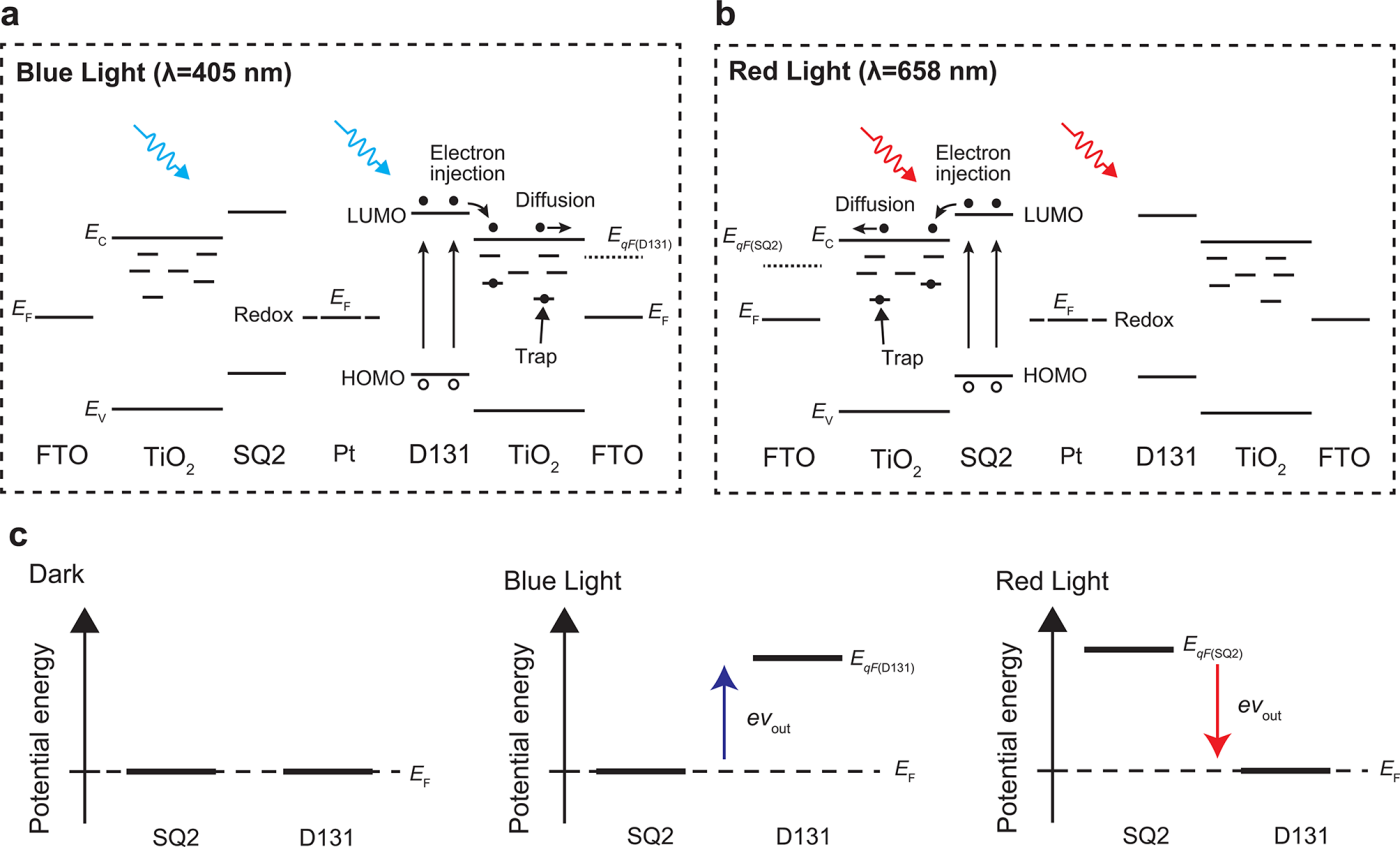
Mechanism of the wavelength-dependent bipolar behavior in the device. (**a**) Band diagram of the device under blue light and (**b**) red light irradiation. (**c**) Schematics of the potential energy alignment for TiO_2_ sensitized with D131 and SQ2.

Band diagrams were constructed under red and blue light irradiation to further investigate the mechanism underlying the wavelength-dependent bipolar behavior (Fig. 2a, b). The carrier transport mechanism in DSCs has been well-established in previous studies^34-36^ Electrons were excited from the highest occupied molecular orbital (HOMO) to the lowest unoccupied molecular orbital (LUMO) of the dye molecules under light irradiation. These excited electrons were injected into the conduction band of TiO₂ and subsequently driven by diffusion. *E*_c_ and *E*_v_ represent the energy level of the conduction band minimum and the valence band maximum, respectively. The electrons were accumulated in TiO₂, leading to an increase in the quasi-Fermi level.

Under blue light irradiation, only D131 absorbed light, increasing the quasi-Fermi level in TiO₂ sensitized with D131 (*E*_qF(D131)_). However, only SQ2 absorbed light under red light irradiation, increasing the quasi-Fermi level of TiO₂ sensitized with SQ2 (*E*_qF(SQ2)_). Under open-circuit conditions, the potentials of the counter and working electrodes were equivalent to the redox level of iodide/triiodide and the Fermi level (*E*_F_) of TiO₂, respectively^37^ Consequently, *v*_out_ reflects the difference in the energy levels between the TiO₂ electrodes sensitized with D131 and SQ2.

Figure 2c presents a schematic of the potential energy alignment for TiO₂ sensitized with D131 and SQ2. *E*_qF(D131)_ and *E*_qF(SQ2)_ were equal to *E*_F_ under dark conditions, resulting in a *v*_out_ of 0 V. Upon blue light irradiation, *E*_qF(D131)_ exceeded *E*_qF(SQ2)_, producing a positive *v*_out_. However, *E*_qF(SQ2)_ surpassed *E*_qF(D131)_ under red light irradiation, yielding a negative *v*_out_.

The origin of the bipolar behavior can be attributed to the distinct responses of the different dyes to light of varying wavelengths. This approach facilitates the design of artificial optoelectronic synapses that produce both positive and negative voltages by selectively choosing the appropriate dyes. Consequently, optoelectronic synapses with bipolar behavior across different wavelengths in the visible spectrum can be effectively executed.

**Fig. 3:**
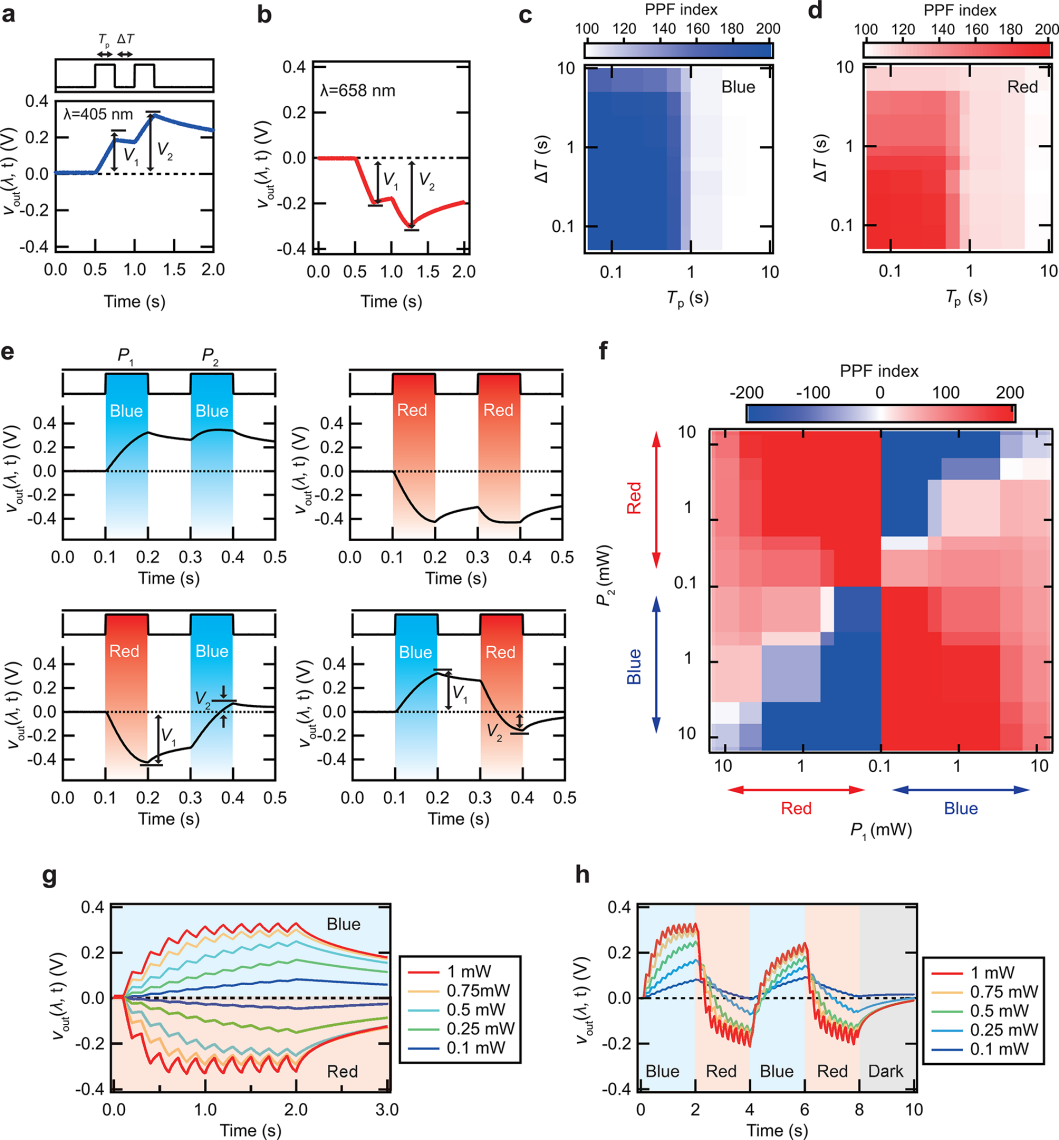
Synaptic properties of dye-sensitized optoelectronic artificial synapse. (**a**) Typical *v*_out_ response induced by two successive blue light pulses and (**b**) red light pulses (*T*_p_ = 250 ms, Δ*T* = 250 ms, *P* = 0.425 mW, λ = 405, 658 nm). (**c**) Average PPF index as a function of *T*_p_ with varying Δ*T* under blue light illumination and (**d**) red light illumination (*T*_p_ = 0.05–10 s, Δ*T* = 0.05–10 s, *P* = 0.425 mW, λ = 405, 658 nm). **(e**) Typical *v*_out_ response induced by blue and red light irradiation (*P* = 5 mW, λ = 405, 658 nm). (**f**) Average PPF index induced by blue and red light irradiation as a function of light intensity (*P* = 0.25–15 mW, λ = 405, 658 nm). (**g**) Dependence of the *v*_out_ response on light intensity, induced by 10 successive pulses (*T*_p_ = 0.1 s, Δ*T* = 0.1 s, *P* = 0.1–1 mW, λ = 405, 658 nm). (**h**) *v*_out_ response under input wavelengths, changing every 10 successive pulses (*T*_p_ = 0.1 s, Δ*T* = 0.1 s, *P* = 0.1–1 mW, λ = 405, 658 nm).

We examined the synaptic properties of the device to assess its ability to process time-series data. One such property, the paired-pulse facilitation (PPF) index, was measured. The PPF index is defined as the ratio of the output voltages induced by two successive pulses, as shown below:

$$\:\begin{array}{c}\rm{PPF\:index}=\frac{{V}_{2}}{{V}_{1}}\times\:100,\:\left(2\right)\end{array}$$

where *V*_1_ and *V*_2_ correspond to the voltages induced by the first and second light pulses, respectively (see Fig. 3a, b).

Figure 3a presents the typical response of *v*_out_ induced by two successive blue light pulses (λ = 405 nm) using a laser. The pulse width (*T*_p_) and the interval (Δ*T*) were both set to 250 ms. The PPF index exceeded 100% for *V*_2_ > *V*_1_, indicating facilitation. Similarly, Fig. 3b illustrates the response of *v*_out_ induced by two successive red light pulses (λ = 658 nm). In this case, even though *V*_2_ is smaller than *V*_1_, the PPF index still exceeded 100%. Regardless of whether *v*_out_ is positive or negative, the PPF index surpassed 100% if *V*_1_ and *V*_2_ share the same sign and ∣*V*_2_∣>∣*V*_1_∣, indicating facilitation. Figure 3c, d displays the PPF index as a function of *T*_p_ for various Δ*T* values. Under blue light, the PPF index increased as *T*_p_ and Δ*T* decreased, reaching a peak of 231%. This trend aligns with previous reports^28^ Similarly, the PPF index reached 202% in the same region under red light. These results demonstrate that the device exhibited synaptic responses induced by both red and blue light pulses.

Artificial synapses must reset their excited states to the initial condition to sequentially process time-series data. Typically, this requires a waiting period corresponding to the relaxation time, which limits continuous operation. Alternating the light wavelengths between red and blue may facilitate a faster reset of *v*_out_ to 0 V since blue and red light induce opposite polarities of *v*_out_, potentially bypassing the need for a full relaxation period.

We measured the PPF index by varying the wavelengths of the first and second light pulses to investigate this reset mechanism. The light intensities of the first and second pulses, denoted as *P*_1_ and *P*_2_, were controlled. The typical *v*_out_ response is shown in Fig. 3e (*P*_1_, *P*_2_ = 0.425 mW, *T*_p_ = 100 ms, Δ*T* = 100 ms). Applying two consecutive pulses of the same wavelength resulted in ∣*V*_2_∣>∣*V*_1_∣, indicating facilitation. Notably, when the wavelengths of the first and second pulses were alternated, *v*_out_ quickly returned to 0 V after the second pulse, suggesting that alternating wavelengths effectively reset *v*_out_ faster than the relaxation time.

Figure 3f presents the average PPF index induced by blue and red light pulses at varying light intensities (*P*_1_, *P*_2_ = 0.25 − 15 mW, λ = 405, 658 nm). As reported in previous studies, applying two consecutive pulses of the same wavelength resulted in facilitation (*P*_2_ > *P*_1_, PPF index > 100%) or depression (*P*_2_ < *P*_1_, PPF index < 100%) in DSCs^28^ When the wavelengths of the first and second pulses were changed, the PPF index varied between positive and negative values. At approximately *P*_1_ = *P*_2_, the PPF index approached zero, confirming the reset function. When *P*_1_ > *P*_2_, the PPF index ranged from 0 to 100, indicating that the second pulse reset *v*_out_ toward 0 V without fully returning it to 0. Conversely, when *P*_1_ < *P*_2_, the PPF index became negative, signifying that *v*_out_ reached 0 V during the second pulse and then reversed polarity beyond 0 V. This demonstrates that alternating light wavelengths can reset the device more rapidly than the intrinsic relaxation time.

Typically, the PPF index of artificial synapses ranges from 100 to 200^38^ However, surprisingly our device demonstrated an exceptionally broad PPF index, spanning from − 3776 to 8075. This behavior is attributed to the sensitivity of *v*_out_ to both light intensity and wavelength, which enables polarity switching. In PRC applications, generating distinct outputs for various inputs is crucial^39^ Consequently, the expansive range of PPF indices observed in our device highlights its potential for processing time-series data involving variations in both light intensity and wavelength.

Following that, we measured the *v*_out_ responses under light pulse irradiation while varying both light intensity and wavelength. Figure 3g illustrates the *v*_out_ responses induced by ten successive pulses of red and blue light at varying intensities. Blue light pulses resulted in positive facilitation, while red light pulses induced negative facilitation, reflecting the bipolar synaptic behavior of the device. Figure 3h shows the *v*_out_ responses to 40 successive pulses, with the wavelength alternating every 10 pulses. Following positive facilitation under blue light, red light irradiation induced a depression toward 0 V, followed by negative facilitation. Similarly, blue light irradiation led to a depression toward 0 V after facilitation under red light, followed by positive facilitation. The responses of the *v*_out_ changed clearly with light intensity. The time-series data involving light intensity and wavelength generated unique *v*_out_ responses, confirming that the device can function as a PRC for time-series data for both light intensity and wavelength. We conducted time-series data processing tasks, including classification and logic operations, based on variations in light intensity and wavelength to explore the practical application of this PRC.

**Fig. 4:**
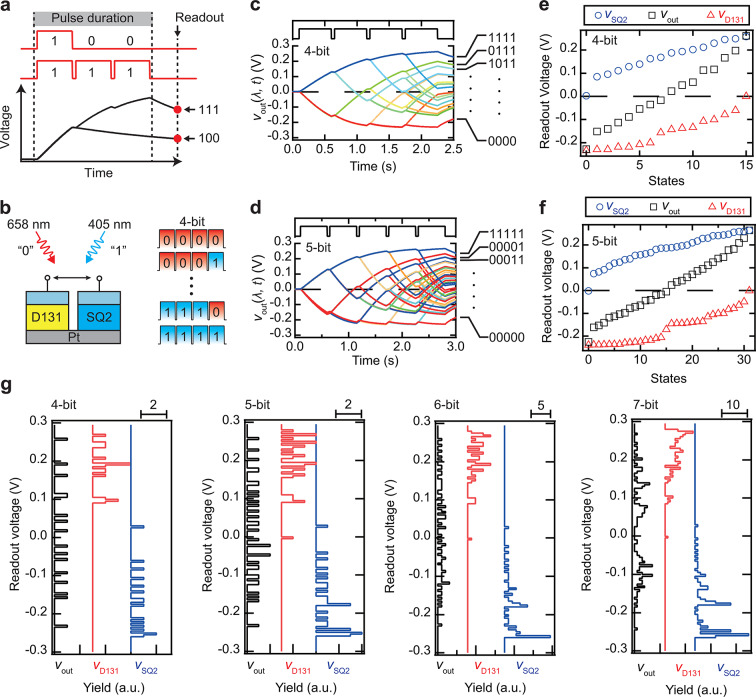
Classification performance of the DSC-based bipolar artificial synapse in physical reservoir computing. (**a**) Schematic representation of the readout value concept. (**b**) Schematic diagram of the experimental setup. (**c**) Transient voltage response induced by 4-bit and (**d**) 5-bit optical pulses (*T*_p_ = 500 ms, Δ*T* = 50 ms, *P* = 0.5 mW). (**e**) Readout voltage values as a function of 4-bit and (**f**) 5-bit states (Readout time = 10 ms). (**g**) Histogram of the readout voltage for 4-bit to 7-bit operations.

Classification tasks, such as handwritten digit or fingerprint classification, serve as benchmark tasks for assessing the computational performance of PRCs^9,40^ A key aspect of these tasks is the concept of readout voltage. Figure 4a illustrates the concept of the readout voltage in a classification task. The readout voltage is defined as the voltage value measured after a specified readout time following the completion of light-pulse irradiation. The schematic shows two distinct input pulses labeled “100” and “111”. These pulses induce different voltage responses, resulting in unique readout voltage values. If the readout voltage values are indistinguishable, different input pulses will appear identical, rendering the system unsuitable for PRC applications. Overlapping readout values complicate the separation of spatial characteristics, which can impair recognition accuracy^41^ Therefore, the wide range of PPF index values exhibited by our device is anticipated to enhance classification task performance in PRCs by improving the ability to differentiate input patterns more effectively.

We examined the number of input patterns that could be distinguished based on a readout voltage ranging from 4 to 7 bits to assess the classification capabilities of the device. For instance, a 4-bit pulse enables 16 unique input combinations. If the readout voltage can be differentiated into 16 distinct levels, the device can classify 4-bit input pulses. Figure 4b presents a schematic diagram of the experimental setup. We utilized the bipolar synaptic behavior of the device, employing blue light (representing “1”) and red light (representing “0”) for pulse encoding (*P* = 0.5 mW, *T*_p_=500 ms, Δ*T* = 50 ms).

Figure 4c, d presents the transient *v*_out_ responses induced by 4-bit and 5-bit optical pulses, respectively. These transient responses vary according to the input pulse. Figure 4e, f displays the readout voltage as a function of the bit state, with 16 and 32 states for 4-bit and 5-bit operations, respectively. Results from a typical DSCs sensitized with a single dye (SQ2 or D131) are also shown for comparison. In the 4-bit operation, the readout voltage spans from − 0.23 to 0 V for SQ2 and from 0 to 0.26 V for D131. A typical DSC, as reported in the literature, clearly distinguishes 4-bit values^28^ However, the voltage range of *v*_out_ in our device extended from − 0.23 to 0.26 V, broader than that observed in single-dye DSCs. This trend is also evident in 5-bit operations.

Figure 4g presents a histogram of the readout voltage for 4-bit to 7-bit operations. Our device can classify up to 6 bits with a maximum yield of three. However, the maximum yield increased to seven for 7-bit operations, indicating a higher level of difficulty in classification. In contrast, DSCs sensitized with either D131 or SQ2 reached a maximum yield of 4 for 5-bit operations and 6 for 6-bit operations, making classification more challenging. These results suggest that our device offers superior separability compared to typical single-dye DSCs. Previous studies on artificial synapses and PR devices have primarily focused on 4-bit classification, with limited research on 5-bit or higher classifications^41-43^ Therefore, our device demonstrates an enhanced classification capability.

**Fig. 5:**
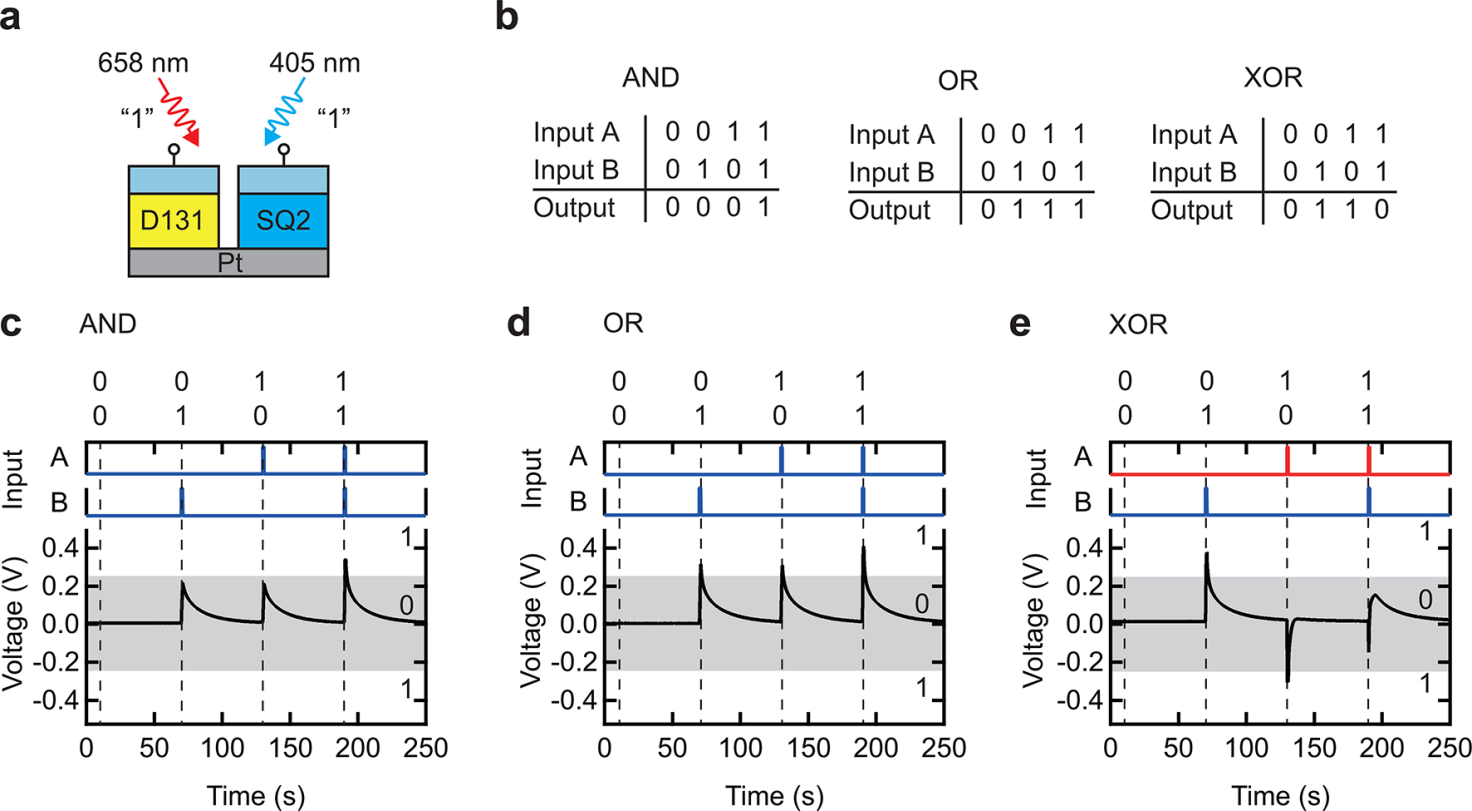
Logic function implementation. (**a**) Schematic diagram of the experimental setup. (**b**) Truth tables for AND, OR, and XOR operations. (**c**) AND operation (*P =* 0.1 mW for both blue light inputs). (**d**) OR operation (*P =* 0.3 mW for both blue light inputs). (**e**) XOR operation (*P =* 0.2 mW for blue light and 2.6 mW for red light).

Logic operations serve as benchmark tasks for artificial synapses to assess their capability in processing time-series data^21,22,26,27,44^ This study demonstrated AND, OR, and XOR operations using red and blue light as inputs. Figure 5a illustrates the experimental setup for these logic operations. Figure 5b shows the truth table for AND, OR, and XOR operations. The output was defined as 0 when ∣*V*∣ < 0.25 V and as 1 when ∣*V*∣ > 0.25 V. Figure 5c, d depicts the results of the AND and OR operations, respectively, which were successfully implemented by modulating the light intensity at 0.1 and 0.3 mW.

Furthermore, XOR—a nonlinear operation—was demonstrated by varying the wavelength of incident light by exploiting the unique bipolar characteristics of the device (Fig. 5e). XOR functionality was achieved using both red and blue light as inputs, with intensities of 2.6 and 0.2 mW, respectively.

Conventional unipolar photodetectors can perform basic logic operations such as AND and OR; however, implementing more complex operations like XOR, NAND, and NOR remains challenging^45,46^ In contrast, the proposed device enables not only AND and OR operations but also the more complex XOR function due to its ability to modulate responses based on light intensity and switch polarity with wavelength variations. These findings demonstrate the capability of the device for processing time-series data across different light intensities and wavelengths, facilitating the execution of advanced logic operations. Such characteristics make it particularly suitable for applications in optical sensors that use multiple wavelengths, including blood-oxygen saturation monitoring^47^ and heartbeat signal detection^12^.

However, there remain issues that must be addressed for practical applications. This is because the logic level determination is currently based on the condition |V| < 0.25 V for logical “0” and |V| > 0.25 V for logical “1”. In the future, we envision obtaining the output shown in Fig. 5b by passing the device’s output voltage through a bridge circuit composed of rectifying devices with low threshold voltages (approximately 0.25 V), such as Schottky diodes. In addition, although the output in this demonstration exhibited values close to the boundary between logical “0” and “1”, we believe that the successful implementation of AND, OR, and XOR logic functions is meaningful as a proof of concept. For practical use, it will be necessary to design devices that generate output voltages capable of clearly distinguishing between “0” and “1”.

A classification task was performed on time-series data of light intensity and wavelength using a PRC system to demonstrate the capability of the device. Conventional video capture systems rely on at least three separate photodiodes (PDs) equipped with red, green, and blue filters. The outputs from these photodiodes are combined to generate a color video. However, this approach presents several limitations, including the need for many photodiodes and complex electronic circuitry.

**Fig. 6:**
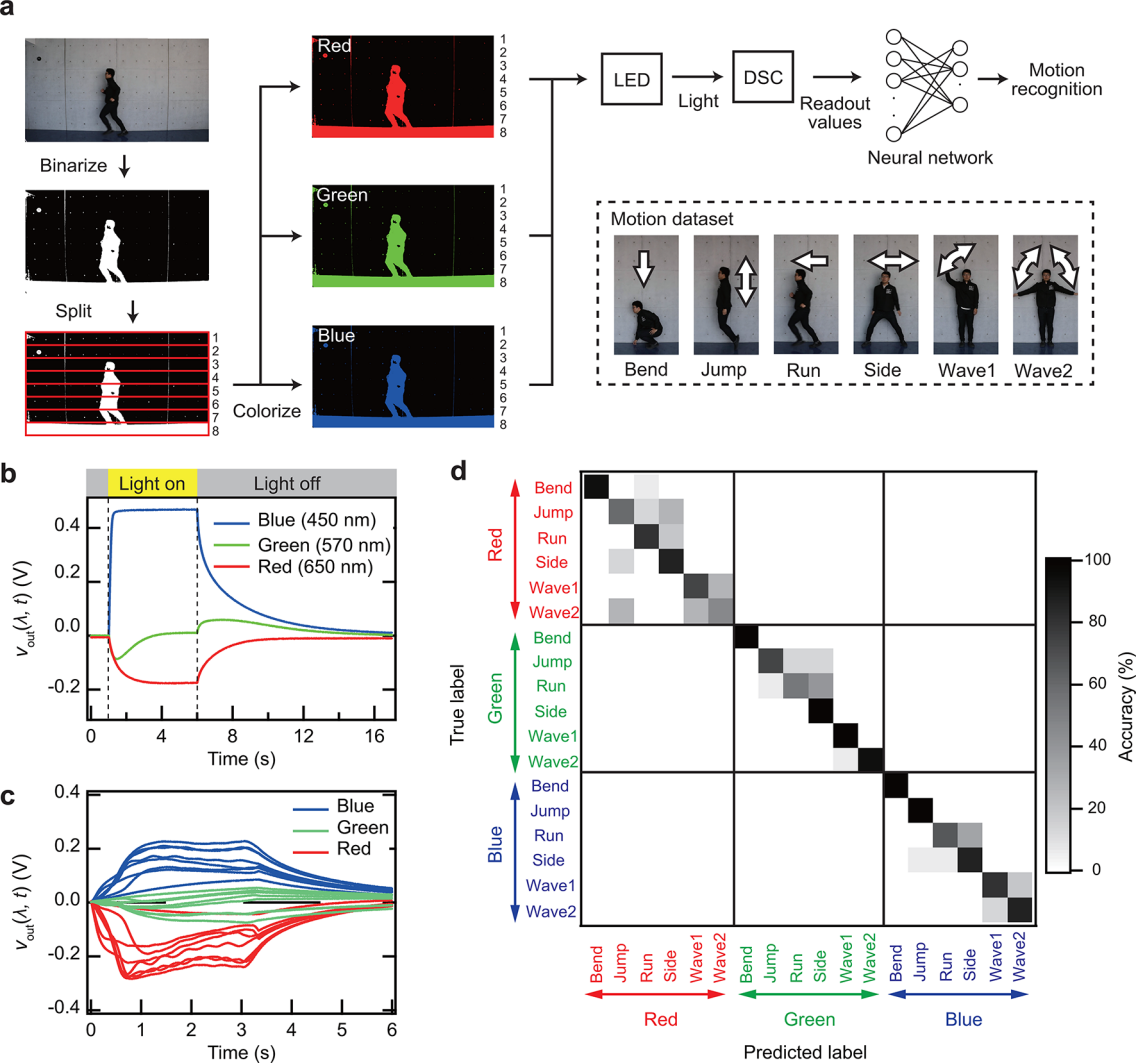
Demonstration of multicolor motion-recognition task using a PRC system with the self-powered polarity-tunable dye-sensitized optoelectronic artificial synapse. (**a**) Schematic representation of the multicolor motion-recognition task. (**b**) Transient voltage response of the device induced by blue, green, and red light pulses (blue: λ = 450 nm, green: λ = 570 nm, red: λ = 650 nm). (**c**) Transient voltage response of the device under blue, green, and red light irradiation (λ = 415, 570, and 625 nm) for the jump action. (**d**) Confusion matrix for the multicolor motion recognition task.

The proposed device exhibited distinct *v*_out_ responses: positive for red light, negative for blue light, and near-zero for green light. These response characteristics suggest its potential applicability in color video recognition systems with a single device. Hence, a multicolor motion-recognition task was conducted to investigate this possibility (see Fig. 6a). Six different actions—bending (bend), waving with one hand (wave1), waving with both hands (wave2), jumping (jump), running (run), and moving sideways (side)—were recorded as video sequences and binarized. Each video was divided into eight segments and converted into red, green, or blue signals. These segments were sequentially input into the DSC device using LEDs with wavelengths of 650 nm (red), 570 nm (green), and 450 nm (blue). Multicolor motion-recognition task was performed based on the readout values obtained from the device, with the readout time set at 10 ms. Further experimental details are provided elsewhere^28^.

Figure 6b presents the *v*_out_ responses induced by red, green, and blue light pulses (*T*_p_ = 5 s). Under blue light illumination, *v*_out_ saturated at 0.47 V in the positive region and decayed toward 0 V after illumination ceased. Under red light, *v*_out_ saturated at -0.17 V in the negative region and similarly decayed toward 0 V upon cessation of illumination. Notably, *v*_out_ initially shifted to the negative region before stabilizing near 0 V under green light exposure. After the green light was turned off, *v*_out_ briefly exhibited a slight positive shift before decaying back to 0 V. These results confirm distinct responses to red, green, and blue light.

Figure 6c illustrates the *v*_out_ responses to the “jump” action under red, green, and blue light irradiation. Similar to the responses observed for individual light pulses, the same motion induced distinct *v*_out_ responses depending on the wavelength of the incident light.

Figure 6d presents the confusion matrix for multicolor motion-recognition task. The classification of the three colors achieved 100% accuracy, while the overall accuracy for motion and color recognition reached 82%. This accuracy is slightly lower than that obtained in our previous work^28^ using a single DSC for the same task. We attribute this to the lower readout voltage in the current device structure compared to the previous report. On the other hand, the ability to achieve perfect color recognition, which was not possible with a single DSC, is a significant advantage. “Bend” showed high classification accuracy for all colors because the video was divided into horizontal strips, which would make vertical movements easier to recognize. In contrast, “jump”, “run”, and “side” were often misclassified among themselves, this would be due to their similar horizontal motions. Likewise, “wave1” and “wave2”, both involving stationary hand-waving, were misclassified. This result demonstrates the capability of the device to effectively distinguish between motion and color. The ability to detect red, green, and blue using a single device enables the development of compact, energy-efficient machine vision system, such as optical sensors for autonomous vehicles. These sensors need to classify various colorful time series data, including traffic light, road signs, pedestrians, and animals while minimizing power consumption. Our findings contribute to energy-efficient classification of time-series color data in such scenarios.

The distinct responses to red, green, and blue light originate from the absorption characteristics of the SQ2 and D131 dyes and their crossover effects, as previously discussed. Tailoring absorption wavelengths and their crossover facilitates the design of three distinct color responses owing to their dependence on dye properties. This discovery paves the way for developing self-powered, polarity-tunable, wavelength-dependent dye-sensitized optoelectronic artificial synapses achieving color recognition.

## Conclusion

This study introduced a self-powered, polarity-tunable artificial synapse by integrating DSCs. The device could distinguish wavelengths with a resolution of 10 nm, demonstrating distinct voltage responses: a positive *v*_out_ under blue light and a negative *v*_out_ under red light. These wavelength-dependent responses stem from the selective absorption characteristics of the two DSCs. The synaptic responses to red and blue light resulted in a PPF index ranging from − 3776 to 8075, significantly broader than those reported for conventional artificial synapses. This enhanced dynamic range enabled the device to distinguish up to six-bit input patterns, surpassing the classification capability of standard DSCs. Furthermore, the device successfully implemented logic operations, including AND, OR, and XOR, owing to its dual functionality of response modulation based on light intensity and polarity switching based on wavelength.

As a proof of concept, the device functioned as a physical reservoir and achieved 82% classification accuracy in a task involving three colors (red, green, and blue) and six distinct human motions. The proposed integration approach enables wavelength-specific response engineering by selecting dyes, paving the way for self-powered artificial synapses capable of high-precision wavelength discrimination.

## Methods

### Device fabrication

A FTO glass substrate (2.5 cm × 2.5 cm, sheet resistance: 7 Ω/□, NPV-CFT2-7 C, AS ONE CORPORATION, Japan) was sequentially cleaned with acetone, ethanol, and deionized water. A nanoporous TiO₂ layer was deposited onto the FTO substrate using the doctor blade method with TiO₂ paste (PST-18NR, JGC Catalysts and Chemicals Ltd., Japan). Detailed fabrication procedures have been described previously^28^ Two TiO₂ electrodes were prepared by immersing them in different dye solutions. One electrode was soaked in a 0.1 mM solution of a squarylium derivative-based dye (SQ2, Solaronix S.A., Aubonne, Switzerland) for 24 h, while the other was immersed in a 0.3 mM solution of an indoline-based dye (D131, 797391, Sigma-Aldrich) for the same duration. After that, the dye-sensitized TiO₂ electrodes were assembled using a thermoplastic spacer (HIMILAN, Dow-Mitsui Polychemicals Company, Ltd., Japan) and filled with a 0.15 M tri-iodide electrolyte solution (Z-150, Solaronix S.A., Aubonne, Switzerland). A platinum plate (2.5 cm × 2.5 cm) was affixed as the counter electrode. The active electrode area was typically 0.8 cm².

### Device characterization

IPCE spectra were recorded using a Peccell Technologies S10AC system with a 150 W xenon lamp. The measurement step interval was set to 5 nm with a delay of 2 s. Electrical measurements were conducted using a DAQ device (USB-6366, National Instruments, TX, USA). The wavelength dependence of *v*_out_ was analyzed using a spectrometer (SM-560, JASCO Corporation, Japan). The voltage responses of individual dye-sensitized electrodes, *v*_SQ2_ and *v*_D131_, were measured separately on masked regions. Synaptic properties, classification performance, and logic operations were evaluated by irradiating the DSCs with red and blue laser diodes (λ = 658 nm and 405 nm, L658P040, L405P20, THORLABS Inc., USA). Light intensity was modulated using a diffuser to ensure uniform irradiation across the device, with radiation power ranging from 0.1 to 10 mW. All experiments were conducted at ambient pressure and room temperature.

### Motion recognition task

The motion recognition dataset was generated by our research group and is not publicly available. All individuals included in the training dataset are members of our research team who participated voluntarily in the recordings. Some of them are co-authors of this study, while others are not listed as authors. As no external participants or personally identifiable information were involved, and no interventions or behavioral evaluations were conducted, ethical approval was not required for this study. The individual appearing in Fig. 6 is a co-author of this study (H.K.), who voluntarily agreed to appear in the figure for demonstration purposes. Informed consent was obtained for the publication of the image in this open-access journal. Since the image was used solely for illustrative purposes and involved no experimental procedures, institutional ethics approval was not required. Motion recognition datasets were collected for six distinct actions: bend, wave1, wave2, jump, run, and side. The bend, wave1, and wave2 actions were performed in a stationary position at the center of the screen, whereas jump, run, and side involved lateral movement across the screen.

Motion data were captured using a commercial camera (EOS Kiss X9, Canon Inc., Japan) at a frame rate of 60 FPS. The video duration changed slightly depending on the action. Subsequently, each video was converted into a binary format at 30 FPS and divided into eight horizontal strips. The video frames were colorized in red, green, and blue.

Time-series data for light intensity were obtained by computing the average luminance *P*_ave_ from *P*_(x, y,t)_, representing the luminance of each pixel within a strip. Motion features were extracted using the expression $\:\frac{{P}_{\text{a}\text{v}\text{e}}\left(t\right)-{P}_{\text{m}\text{i}\text{n}}}{{P}_{\text{m}\text{a}\text{x}}}$, where *P*_min_ and *P*_max_ denote the minimum and maximum luminance values, respectively. The processed time-series data were input into an LED system, and the transient voltage response was recorded. Following that, the colorized time-series data were irradiated onto the DSC device using LEDs at wavelengths of 625 nm (red), 570 nm (green), and 415 nm (blue). The *P*_max_ values for red, green, and blue were set to 0.6 mW, 0.3 mW, and 0.1 mW, respectively. The pulse width (*T*_p_) was fixed at 0.033 s to maintain the motion speed, with the video endpoint defined as a readout time of 0 s. The readout time was set to 10 ms.

A simple neural network with a single layer was used to process the data. The Keras open-source library was utilized for NN implementation. The network employed a cross-entropy loss function with a 1 × 8 input layer, a 1 × 18 output layer, and no hidden layers. The Softmax function was used as the activation function, while RMSprop was employed as the optimizer. The video database included 24 videos per person for each action and each color. In total, 171 videos per action were used for training, while 45 videos were allocated for testing. Further methodological details are provided in a previous study^28^.

## Electronic supplementary material

Below is the link to the electronic supplementary material.

Supplementary Material 1

## Data Availability

The datasets used and/or analyzed in this study are available from the corresponding author upon reasonable request.
