# Supplementary material for: Polarity-tunable dye-sensitized optoelectronic artificial synapses for physical reservoir computing-based machine vision
Source: Sci Rep. 2025 May 12;15:16488. doi: 10.1038/s41598-025-00693-0 (PMC12069640; doi:10.1038/s41598-025-00693-0)
Supplement: Supplementary file 1 — Supplementary Material 1 [file 41598_2025_693_MOESM1_ESM.docx]

**Supplementary Information**

**Polarity-Tunable Dye-Sensitized Optoelectronic Artificial Synapses for Physical Reservoir Computing-based Machine Vision**

*Hiroaki Komatsu, Norika Hosoda, and Takashi Ikuno**

Department of Applied Electronics, Graduate School of Advanced Engineering,

Tokyo University of Science, Katsushika, Tokyo 125-8585, Japan

*Email: [tikuno@rs.tus.ac.jp](mailto:tikuno@rs.tus.ac.jp)

**
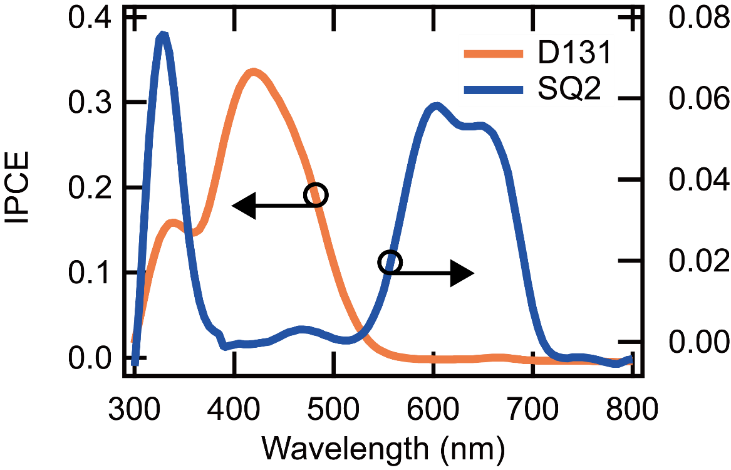
**

**Figure S1.** IPCE spectra of DSCs sensitized with SQ2 and D131.


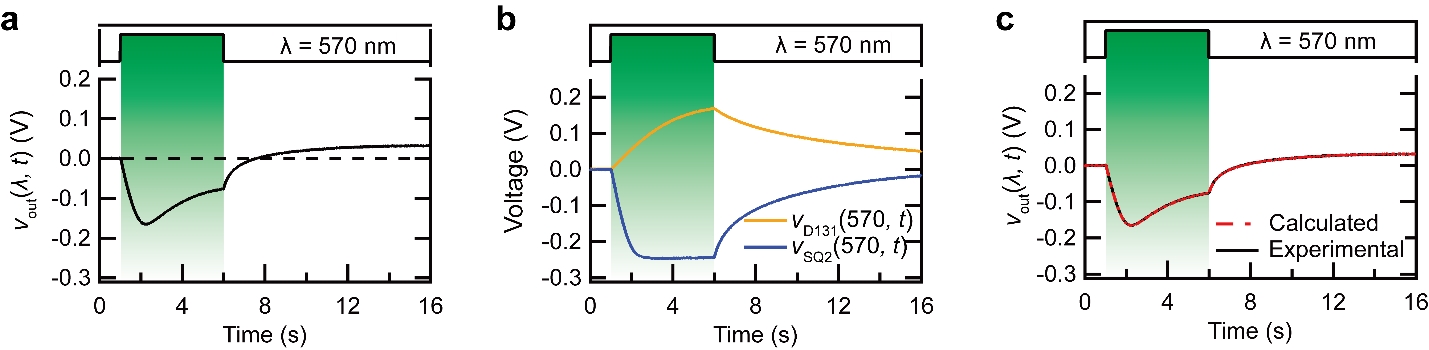
**Figure S2.** (**a**) Transient *v*_out_ response induced by a 570 nm light pulse. (**b**) Transient voltage response of DSCs masked with SQ2 and D131 under a 570 nm light pulse. (**c**) Transient *v*_out_ response and corresponding theoretical calculation based on Equation (1).
